# Quality improvement interventions to prevent neonatal necrotizing enterocolitis: a systematic review

**DOI:** 10.3389/fped.2025.1519029

**Published:** 2025-05-23

**Authors:** Xueli Zhang, Mingqiu Chen, Yanfei Zhang, Jieer Zhou, Tingyan Wei, Zhangbin Yu, Yuqin Yan, Zhangxing Wang

**Affiliations:** ^1^ Division of Neonatology, People’s Hospital of Longhua, Shenzhen, Guangdong, China; ^2^ Department of Neonatology, Shenzhen People’s Hospital, The Second Clinical Medical College, Jinan University, Shenzhen, Guangdong, China; ^3^ The First Affiliated Hospital, Southern University of Science and Technology, Shenzhen, Guangdong, China

**Keywords:** quality improvement, necrotizing enterocolitis, preterm infants, low-birth-weight infants, meta-analysis

## Abstract

**Background:**

Neonatal necrotizing enterocolitis (NEC) is the leading cause of death due to gastrointestinal disease in preterm neonates. Quality improvement bundles could reduce the incidence of NEC in preterm infants, but their replication in neonatal intensive care units has had inconsistent outcomes.

**Objective:**

Quality improvement may reduce the incidence and severity of NEC in preterm infants. We evaluated quality improvement interventions (QIIs) that sought to prevent or reduce the severity of NEC.

**Methods:**

PubMed, Embase, Cochrane Library, Web of Science, Wanfang Database, China National Knowledge Infrastructure (CNKI), VIP Chinese Journal Service Platform (VIP), Chinese BioMedical Literature Database (CBM), and citations of selected articles were searched. QIIs that reduced the incidence or severity of NEC in preterm infants were the primary outcome. Paired reviewers independently extracted data from selected studies.

**Results:**

In total, 13 quality improvement interventions involving 17,961 infants were included. Nearly all of the QIIs included improving breastfeeding rates. Moreover, 16 of the 19 QIIs resulted in a significant reduction in the incidence of NEC after their implementation. Application of the quality criteria of the quality improvement showed that all the interventions were considered to be of medium to high quality, with the lowest score being 8 and 13 of the interventions having scores more than 10. The studies had heterogeneity with significant variations in intervention characteristics, implementation units, personnel, sample size, time, and outcomes.

**Conclusion:**

QIIs resulted in reductions in the incidence and severity of NEC in preterm infants in some but not all settings. The specific interventions and quality improvement methods that were responsible for those reductions and why they were successful in some settings but not others are unclear. This systematic review can assist teams in identifying potentially better practices for reducing NEC.

**Systematic Reviews Registration:**

https://www.crd.york.ac.uk/PROSPERO/view/CRD42024601939, PROSPERO (CRD42024601939).

## 1 Introduction

Neonatal necrotizing enterocolitis (NEC) is a fatal gastrointestinal emergency affecting newborns, especially premature infants. Although the incidence of NEC has declined over time, it still affects nearly 5% of very preterm or very-low-birth-weight infants (1). The mortality rate associated with NEC is 20%, and for cases requiring surgical intervention, this rate can exceed 30% (2). Infants diagnosed with NEC often have limited nutritional intake, delayed extrauterine growth, prolonged hospital stays, increased medical costs, heightened medical needs post-discharge, and an elevated risk for neurodevelopmental disorders (3). Furthermore, when surgical treatment is necessary, NEC can also result in complications such as short bowel syndrome, intestinal insufficiency, liver disease, or the need for organ transplantation (4, 5). These complications not only inflict significant suffering on the child but also impose substantial emotional and financial burdens on families and society.

With the development of evidence-based medicine, evidence-based quality improvement (EBQI) has emerged as a crucial tool for advancing clinical medical practices (6). The use of EBQI can lead to the appropriate use of intervention measures in the neonatal intensive care unit (NICU), which helps to reduce the incidence of neonatal diseases, minimize adverse complications, shorten hospital stays, and lower medical costs (7). Recent studies have shown that quality improvement (QI) measures are effective in reducing NEC; however, the implementation of QIs varies in NICUs across different countries and regions, and there are no systematic and comprehensive interventions utilized in clinical settings.

Patel et al. (8) previously reviewed the impact of quality improvement interventions (QIIs) on NEC; however, the number of studies included in that review was limited, and several new studies have since been published. Therefore, this study presents a systematic review of existing NEC-related interventions, integrating and systematically evaluating their effectiveness and sustainability. The aim is to assist medical teams in analyzing, evaluating, and implementing multi-faceted interventions to prevent and reduce the incidence of surgical necrotizing enterocolitis (SNEC).

## 2 Methods

### 2.1 Registration

This study conforms to the Preferred Reporting Items for Systematic Reviews and Meta-Analysis (PRISMA) consensus guidelines (9) (Supplementary Table S1). The study has been registered with the International Prospective Register of Systematic Reviews (PROSPERO; registration number: CRD42024601939). The systematic evaluation, being a secondary analysis of existing literature, was deemed not to require ethical review.

### 2.2 Data sources and search strategy

We conducted a comprehensive search across multiple databases, including PubMed, Embase, Cochrane Library, Web of Science, Wanfang Database, China National Knowledge Infrastructure (CNKI), VIP Chinese Journal Service Platform (VIP), and Chinese BioMedical Literature Database (CBM). We used the following search strategy for literature (10) to identify literature reports related to NEC: ((quality) AND (improvement *) AND (intervention)) AND (necrotizing enterocolitis) OR (NEC)). In addition, the search terms used included “neonatal necrotizing enterocolitis,” “quality improvement,” and “intervention measures”. Supplementary Table S2 provides a detailed overview of the search strategy. The search encompassed all available literature from the inception of each database until 12 September 2024, without restrictions on language or publication date. In addition, we reviewed references from related studies included in the systematic review.

### 2.3 Eligibility criteria

This study encompasses both single-center and multicenter QIIs, primarily aimed at reducing the incidence and severity of NEC. We conducted a search for studies based on inclusion criteria derived from the PICOS framework (11): (1) population (P): premature infants or low-birth-weight infants; (2) intervention (I): active QI interventions aimed at reducing the incidence or severity of NEC; (3) comparison (C): comparisons between infants receiving QI interventions and those who do not; (4) outcome (O): the incidence and serious complications associated with NEC; (5) study design (S): eligible designs were randomized controlled trials (RCTs), controlled before-and-after study (CBA), uncontrolled before-and-after study (UBA), and interrupted time series study (ITS). The exclusion criteria were as follows: (1) studies focusing on populations outside neonatal intensive care; (2) studies with imprecise designs, or those containing duplicate or overlapping data; (3) non-original research, including conference abstracts, clinical trial registries, reviews, systematic reviews, meta-analyses, guidelines, animal experiments, and case reports; (4) studies with incomplete data.

### 2.4 Study selection

All relevant articles were imported into the reference management software EndNote 21 to identify and remove any duplicates. The titles and abstracts of all the selected articles were independently reviewed by two researchers (XZ and MC), and articles that did not meet the criteria were excluded. The full text was reviewed in detail to further exclude studies with irrelevant content. Disagreements between researchers were resolved through discussion in the research group to reach a consensus.

### 2.5 Data extraction

Data extraction for this study was conducted using a standardized Microsoft Excel data extraction form. Two researchers (XZ and MC) independently extracted the data and subsequently compared their findings. Any disagreements were resolved through group discussion or by consulting a third author (ZW) when necessary. Authors of the articles were contacted for additional data or results as needed. The following data were collected: (1) basic information (authors’ names, year of publication, country of study, study period, and study design), (2) characteristics of the target population, (3) interventions included in the QI bundle, and (4) outcomes (the incidence of NEC and SNEC).

### 2.6 Quality and risk of bias assessments

The key components of the QII evaluation encompass an examination of the context and details of the intervention, and the application of the QII process itself. To assess the quality of the QII studies, we employed the valid and reliable QIIs minimum quality criteria set (QI-MQCS) (12). This set includes 16 areas or content categories: organizational motivation, intervention rationale, intervention description, organizational characteristics, implementation, study design, comparator, data source, timing, adherence/fidelity, health outcomes, organizational readiness, penetration/reach, sustainability, spread, and limitations. Each QII study was evaluated across 16 domains, with a score of 1 assigned if the minimum criteria were met and a score of 0 if they were not. The studies were categorized into three quality levels based on their scores: a score of less than 7 indicated low quality, scores between 7 and 10 indicated moderate quality, and scores greater than 10 indicated high quality. Two reviewers (XZ and MC) independently utilized the assessment tool to evaluate the included studies, and any discrepancies in their evaluations were resolved through group consensus.

### 2.7 Statistical analysis

We identified studies with similar study populations and settings or homogeneity in quality improvement projects. A random effects meta-analysis was conducted using Review Manager version 5.3 software and Stata 17.0. The effect measures were expressed as relative risk (RR) for categorical variables, along with 95% confidence intervals (CIs). The statistical heterogeneity of studies was quantified by using the extent of heterogeneity (I^2^) values with assigned values of low, moderate, and high heterogeneity to I^2^ values of 25%, 50%, and 75%, respectively. When there was heterogeneity, we sought the source of the heterogeneity and applied a sensitivity analysis to observe the effect of each study result on the total effect size. *P* < 0.05 was considered significant for heterogeneity. Publication bias was evaluated using a funnel plot and Egger’s test.

## 3 Results

### 3.1 Literature search and study selection

**Figure 1:**
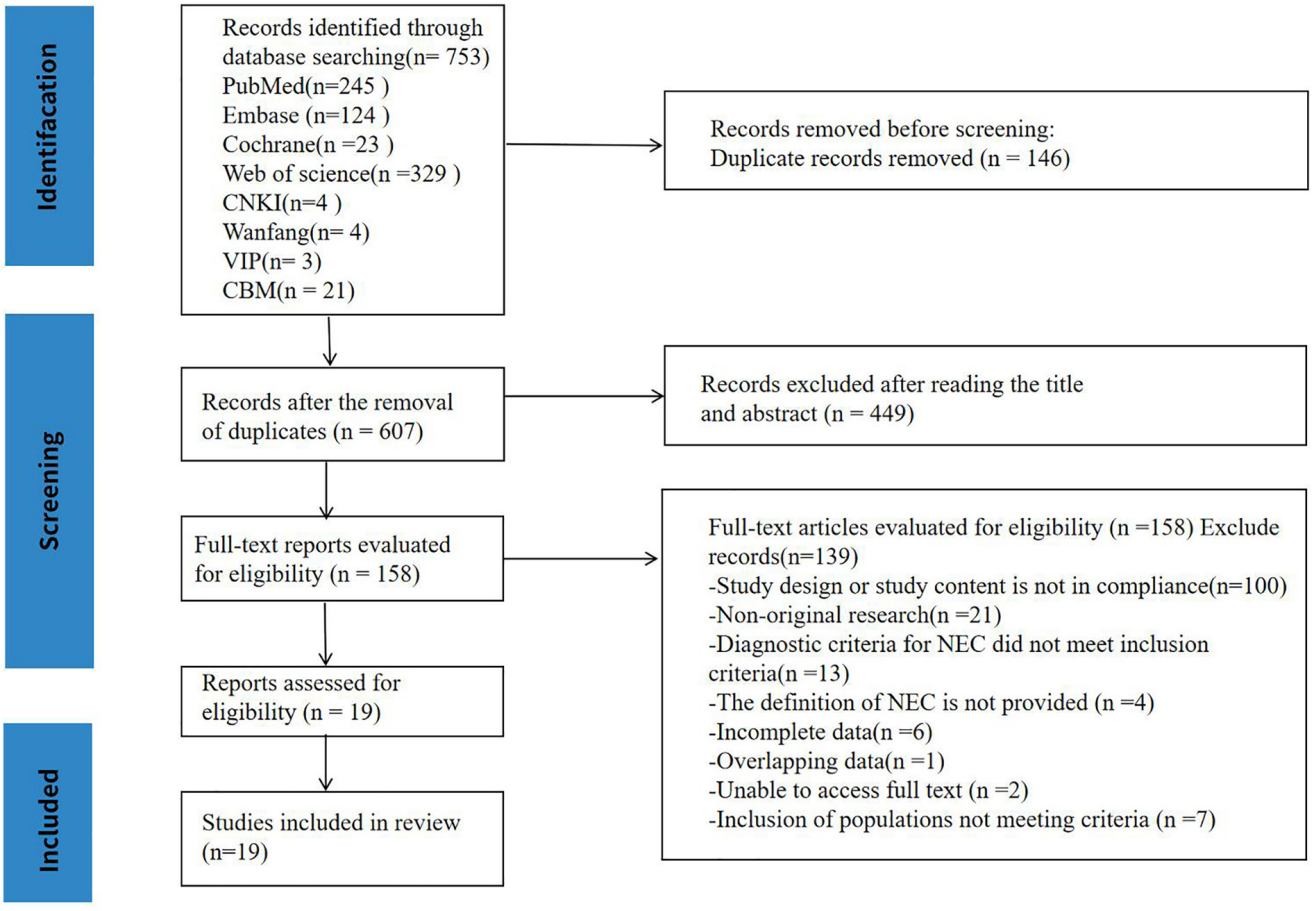
PRISMA flow diagram shows the systematic search of the literature.

We retrieved 753 articles from the database (Figure 1) and excluded 146 duplicates.

Subsequently, 499 articles were eliminated based on the established exclusion and inclusion criteria following a review of titles and abstracts. A total of 158 articles were then screened for full-text review. Ultimately, 139 complete reports were assessed, and after excluding articles with inconsistent design and content, non-original research, and those with incompatible inclusion criteria, incomplete data, or duplicative populations, 19 articles were selected for inclusion. Among the selected studies, 11 (5, 7, 13–21) were QII studies designed with prospective cohorts, while 8 (22–29) were retrospective cohort studies. Among these, 3 (7, 14, 24) were multicenter studies, while the remaining 16 were single-center studies. Only one study (23) included all neonates; almost all the reports utilized gestational age and weight as inclusion criteria. In addition, 11 studies included preterm infants, 12 focused on very-low-birth-weight infants, and 6 studies involved surgical neonates (5, 7, 13, 17, 19, 26).

### 3.2 Characteristics of the included studies

Of the included QIIs, seven took place in the United States, five in Canada, three in China, one in Singapore, and one in Australia, including 17,961 infants in total (Table 1): 6,767 in the pre-intervention group and 11,194 in the intervention group. Intervention inclusion criteria were based on gestational age alone, birth weight alone, or both. Of the 16 QIIs that had exclusion criteria, most excluded infants with congenital anomalies (such as congenital heart disease and gastrointestinal anomalies), premature infants who did not survive, and those born in other institutions.

**Table 1:** Basic characteristics of the included studies.

References	Country, number of institution(s)	Study period	Study population	Size	Study design	Number of interventions	Outcomes
Zhou et al. (13)	China, one NICU	2014–2016	BW < 1,500 g	488	Prospective cohort	5	②
Mavis et al. (5)	USA, one NICU	2019–2022	BW < 1,500 g or GA < 30 weeks	147	Prospective cohort	6	①
Alshaikh et al. (7)	Canada, five NICUs	2013–2021	GA < 32 weeks	2,787	Prospective cohort	5	①
Nathan et al. (14)	USA, three NICUs	2010–2016	BW < 1,500 g	424	Prospective cohort	3	①
Rolnitsky et al. (15)	Canada, one NICU	2015–2018	GA < 33 weeks	1,027	Prospective cohort	3	①
Aziz et al. (22)	Canada, one NICU	2008–2009	28 weeks < GA < 33 weeks	480	Retrospective cohort	1	①
Noonan et al. (23)	USA, one NICU	2007–2018	All infants	NR	Retrospective cohort	5	①
Lee et al. (24)	Canada, 25NICU	2008–2012	GA < 29 weeks	6,026	Retrospective cohort	6	①
Janvier et al. (16)	USA, one NICU	2010–2012	GA < 32 weeks	611	Prospective cohort	2	①
Alshaikh et al. (25)	Canada, one NICU	2009–2011	GA < 32 weeks	443	Retrospective cohort	3	①
Chandran et al. (21)	Singapore, one NICU	2014–2018	BW < 1,500 g	972	Prospective cohort	4	①
Mehtab et al. (17)	USA, one NICU	2014–2018	BW < 1,500 g or GA < 33 weeks	356	Prospective cohort	1	①
Sato et al. (26)	USA, one NICU	2012–2018	BW 1,000–1,499 g	399	Retrospective cohort	1	①
Talavera et al. (18)	USA, one NICU	2010–2013	BW < 1,500 g	941	Prospective cohort	3	①
Patel et al. (19)	USA, one NICU	2008–2012	BW < 1,500 g	451	Prospective cohort	2	①
Sharpe et al. (27)	AUS, one NICU	2007–2014	BW < 1,500 g or GA < 33 weeks	1,791	Retrospective cohort	2	②
Nesterenko et al. (20)	USA, one NICU	2016–2019	BW < 1,500g	408	Prospective cohort	4	②
Chen et al. (28)	China, one NICU	2015–2016	BW < 1,500 g and 25 weeks < GA < 33 weeks	305	Retrospective cohort	1	①
Jing et al. (29)	China, one NICU	2016–2018	BW < 1,500 g and GA < 34 weeks	258	Retrospective cohort	1	①

BW, birth weight; NR, not reported. ① The incidence of NEC decreased significantly; ② The incidence of NEC did not decrease significantly.

Nearly all of the QIIs included improving breastfeeding rates (Table 2), with the exception of three that focused only on either umbilical cord management during delivery (11) or probiotic supplementation (12, 13). The number of clinical interventions in the other QIIs ranged from 1 to 8 or more.

**Table 2:** Interventions included in the QI bundle.

References	（1）	（2）	（3）	（4）	（5）	（6）	（7）	（8）	（9）	（10）	（11）	（12）	（13）
Zhou et al. (13)		**+**											**+**
Mavis et al. (5)		**+**	**+**	**+**		**+**	**+**	**+**	**+**	**+**			
Alshaikh et al. (7)		**+**		**+**	**+**		**+**	**+**	**+**	**+**	**+**		**+**
Nathan et al. (14)	**+**	**+**		**+**					**+**		**+**		
Rolnitsky et al. (15)	**+**	**+**					**+**	**+**					
Aziz et al. (22)	**+**												
Noonan et al. (23)		**+**	**+**	**+**	**+**					**+**		**+**	**+**
Lee et al. (24)		**+**		**+**						**+**			
Janvier et al. (16)							**+**						
Alshaikh et al. (25)		**+**											**+**
Chandran et al. (21)		**+**	**+**				**+**		**+**			**+**	
Mehtab et al. (17)							**+**						
Sato et al. (26)		**+**		**+**			**+**						
Talavera et al. (18)		**+**						**+**		**+**	**+**		
Patel et al. (19)		**+**				**+**							
Sharpe et al. (27)		**+**					**+**						
Nesterenko et al. (20)		**+**			**+**					**+**		**+**	
Chen et al. (28)		**+**											
Jing et al. (29)		**+**	**+**										**+**

QI bundle: (1) Delayed cord clamping; (2) Increase breastfeeding rates; (3) Oral Immune Therapy; (4) Standardized use of breast milk fortification; (5) Standardize management of feeding intolerance; (6) Restriction of residence time of gastric tube; (7) Probiotic supplementation; (8) Restrict the use of H2 blockers and PPI; (9) Standardize antibiotic management; (10) Standardized blood transfusion protocols; (11) Conservative feeding during PDA treatment; (12) Restriction of intestinal nutrient osmotic pressure; (13) Parent education and communication.

Two QIIs included clinical interventions in the delivery room and the implementation of delayed cord clamping (DCC) (11, 14). DCC for 30–60 s when premature infants are born can reduce the incidence of NEC (from 5.4% to 1.3%).

Furthermore, 16 QIIs included clinical interventions to improve breastfeeding rates. Four included creating and training a multidisciplinary mother's milk promotion team (5, 7, 15, 16), 3 focused on providing prenatal breastmilk consultation (7, 12, 15), 1 provided kangaroo care (14), and 2 focused on setting up breast pumping rooms and breast expression guidance (15, 17). Seven QIIs established breastmilk banks and used pasteurized donor milk when breast milk was not available (5, 14, 16, 18–21).

Four QIIs reported oral immune therapy (OIT) (5, 19, 22, 23), which provides drops of fresh colostrum to the oral mucosa to enhance feeding tolerance and prevent NEC.

Six QIIs included the standardized use of milk fortifiers (5, 7, 14, 16, 19, 20), enriching the necessary nutrients and energy of breast milk and thereby addressing the growth and developmental needs of premature infants.

Three QIIs reported standardized feeding intolerance (7, 19, 24), following a weight-specific feeding advancement table from birth until full enteral feeds were tolerated. This involved regular monitoring of gastric residual substances and the amount to develop an individual feeding program.

Two QIIs included limiting gavage tube dwell time (5, 25), leading to the maximal gastric gavage tube dwell time being reduced from 30 to 7 days.

Eight QIIs included supplementation with probiotics (5, 7, 12, 13, 18, 20–22), showing that providing multi-strain probiotics containing *Lactobacillus* and *Bifidobacterium* could reduce the risk of NEC in premature infants and low-birth-weight infants.

Four QIIs reported restricting the use of H2 blockers and proton pump inhibitors (PPIs) (5, 7, 18, 26), as H2 blockers and proton pump inhibitors alter the intestinal bacterial ecology by inhibiting gastric acid secretion and raising pH, increasing the risk of NEC.

Four QIIs included standardizing the use of antibiotics in NICU wards (5, 7, 14, 22), limiting empiric antimicrobial treatment to infants with risk factors for sepsis, and reviewing antimicrobial duration and dosage with laboratory indicators.

Six QIIs included standardized transfusion protocols (5, 7, 16, 19, 24, 26), including implementing standardized thresholds to prevent severe anemia and the avoidance of enteral feeding during transfusion.

Three QIIs reported standardized feeding during patent ductus arteriosus (PDA) treatment (7, 14, 26). Some preterm infants were associated with hemodynamically significant ductus arteriosus (hsPDA), and increasing the feed amount was avoided during treatment for infants with impaired intestinal blood flow and relative intestinal hypoxia. Because ibuprofen is associated with fewer intestinal complications, it is used as a first-line drug in the treatment of PDA.

Three QIIs reported limiting enteral nutrition osmolality (19, 22, 24), and high osmolality of enteral feedings with added medications and supplements in preterm infants has been associated with the etiology of NEC. Therefore, interventions to accurately measure the osmolality of oral medications and supplements to maintain optimal osmolality in the gut are proposed.

Three QIIs also reported education and communication with families (7, 15, 17, 19, 20), including effective communication between healthcare workers and families, access to family breastfeeding support, and encouraging parents of infants to help and support each other as they gain confidence.

### 3.3 Quality assessment

We used the QI-MQCS to assess the quality of the reported intervention content, with higher scores indicating higher quality. Six reports scored 8–10 (15, 16, 21–23, 26), indicating moderate quality; 13 reports scored more than 10 points (5, 7, 13, 14, 17–20, 24, 25, 27–29), indicating high quality. The QI-MQCS scores for each study are shown in Supplementary Table S4.

### 3.4 Meta-analysis

In total, 17 studies (5, 7, 13, 15–22, 24–29) reported the incidence of NEC after the implementation of the intervention, including 18,161 premature or very-low-birth-weight infants with 11,194 infants in the QI group and 6,767 infants in the control group. Two studies (14, 23) did not report enough dichotomous data for us to include them in the meta-analysis. The incidence of NEC decreased significantly in most NICUs after the implementation of QIIs. Specifically, the incidence of NEC decreased after QIIs in the NICU of the Children's Hospital of Fudan University in China (10.00% vs. 7.55%), the NICU of the George Washington University Hospital in Washington, DC (3.5% vs. 5.3%), and the Grantley Stable Neonatal Unit in Australia (2.8 vs. 1.5%), however, there was no significant statistical difference.

**Figure 2:**
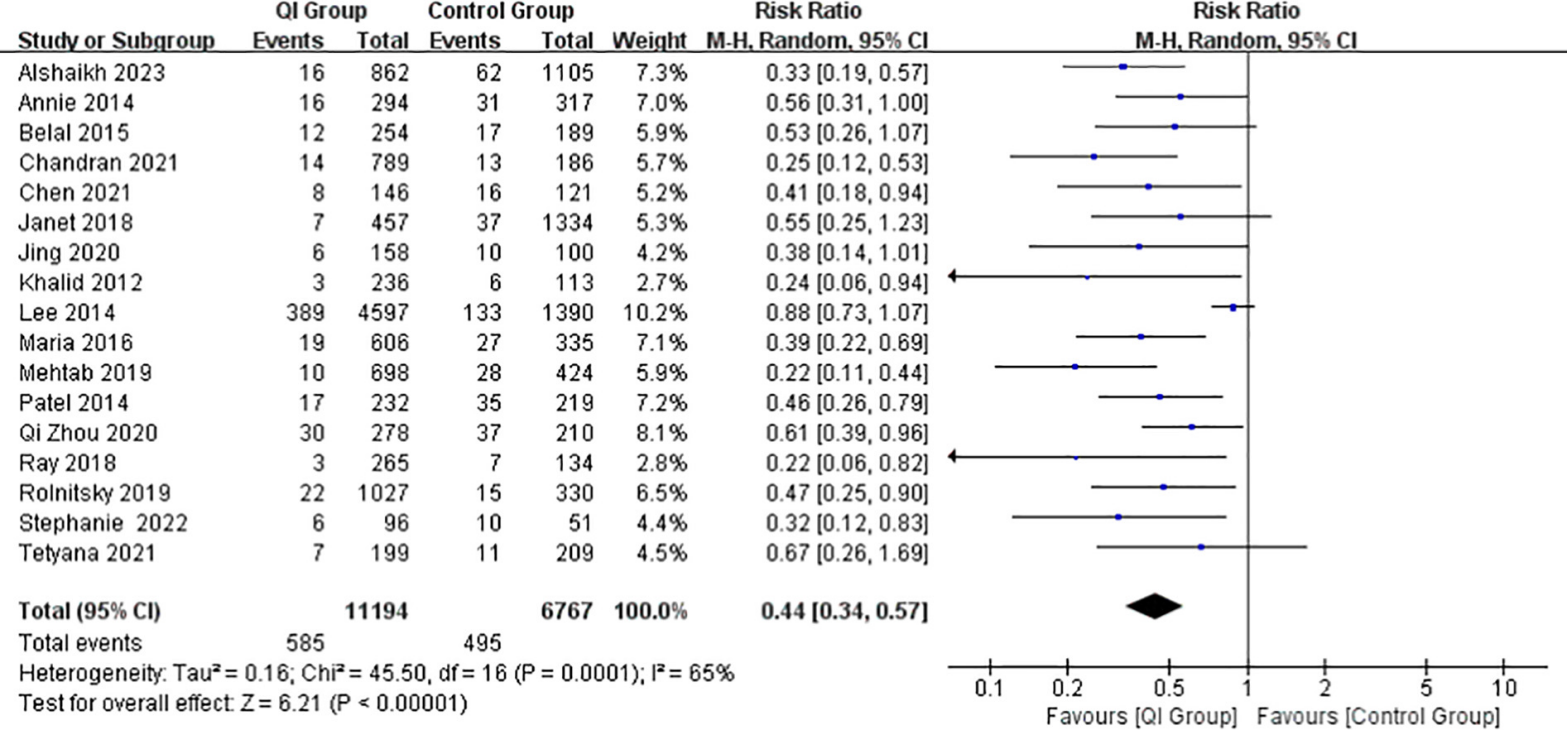
Forest plot from random effects analysis: the rate of NEC before and after QIs.

The combined results showed a significant positive association between QIIs and the incidence of NEC (RR = 2.27, 95% CI: 1.75–2.94, *P* = 0.00001) (Figure 2). Due to the large heterogeneity between the studies (I^2^ = 65%), we examined the impact of each study on the overall risk estimate by excluding one study at a time. When Shoo's study was removed (Supplementary Table S5), the between-study heterogeneity decreased to 0% and the combined RR remained at 2.38 (95% CI: 2–2.82, *P* < 0.00001). The heterogeneity could have been caused by the much larger sample size of this study than the others or by differences in the content of the QI bundles.

**Figure 3:**
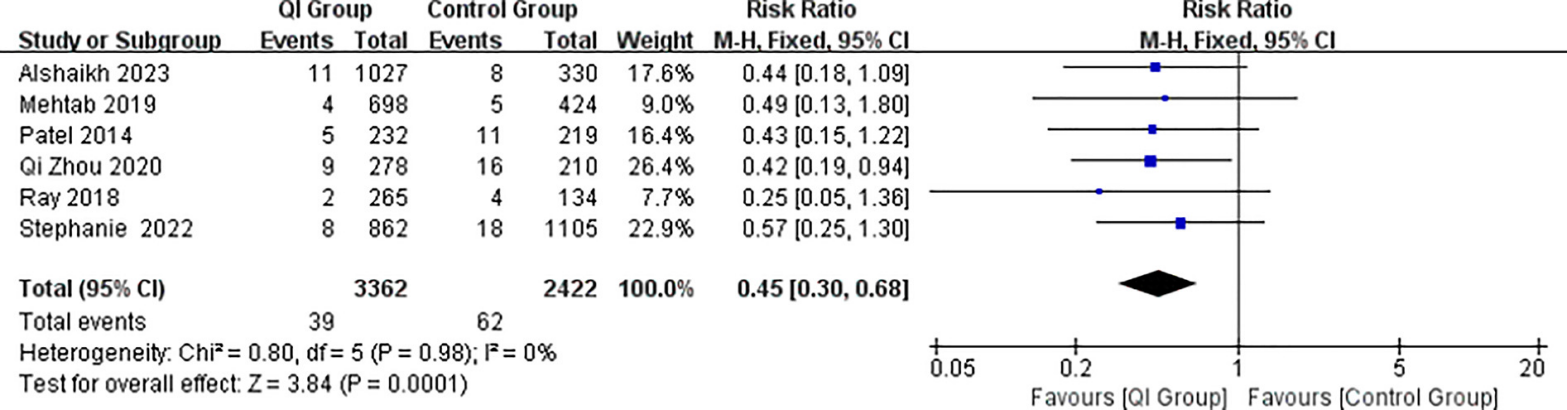
Forest plot from random effects analysis: the rate of SNEC before and after QI.

The meta-analysis showed that the incidence of SNEC was reduced after the implementation of the intervention in six of the studies (Figure 3) (RR = 2.2, 95% CI: 1.47–3.3, *P* = 0.00001), with lower heterogeneity (I^2^ = 0%), suggesting that the QIIs were more effective in reducing surgical NEC.

### 3.5 Subgroup analysis

**Figure 4:**
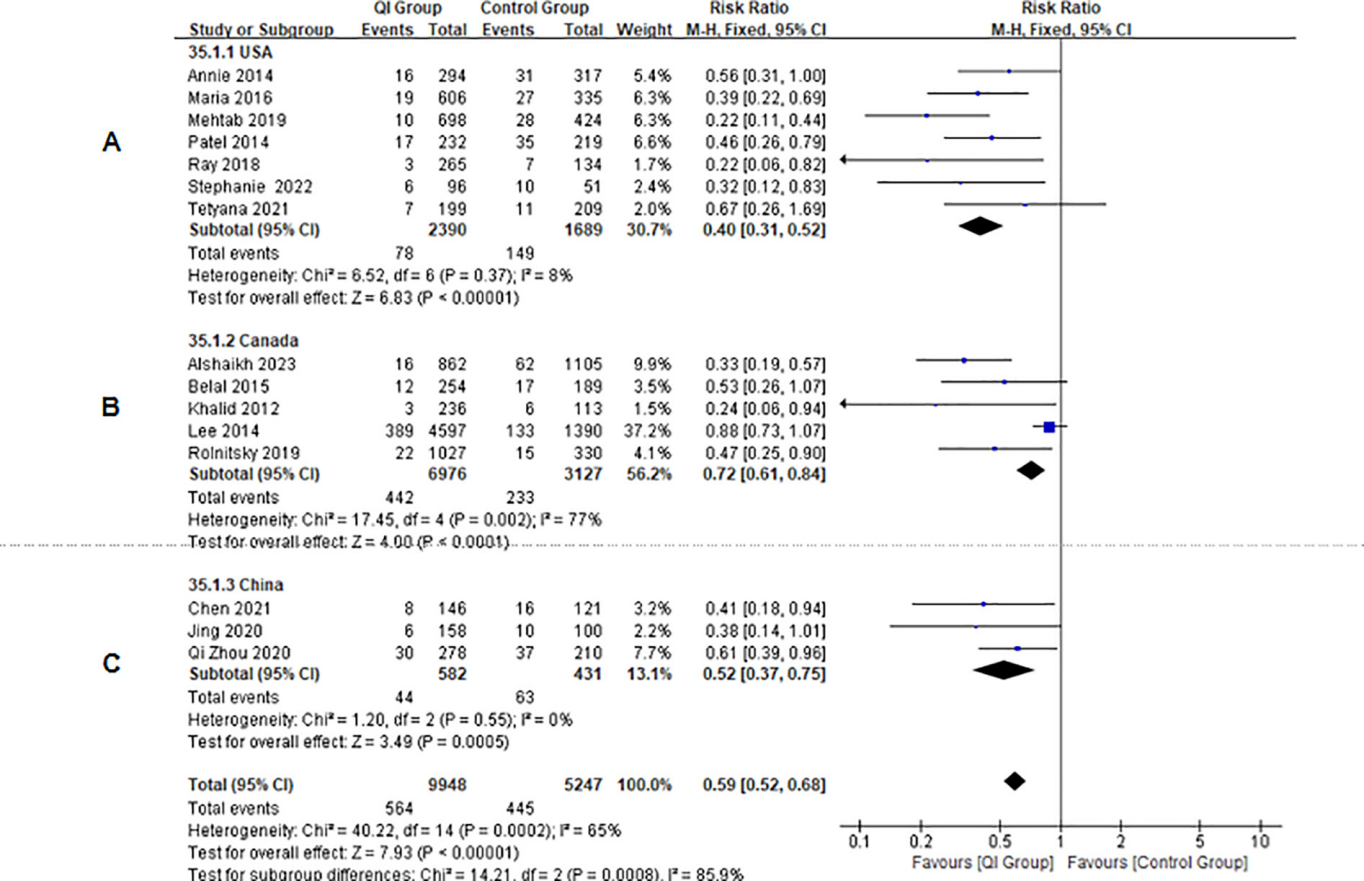
Forest plot from random effects and subgroup analysis: the rate of NEC before-and after QI. **(A)** USA; **(B)** Canada; **(C)** China.

In the subgroup analysis, regardless of the country from which the data came, the incidence of NEC decreased after the QIIs (Figure 4). Subgroup analysis by country showed that the RR in studies from China was 1.89 (95% CI: 1.31–2.71, *P* = 0.0006) and the RR in the studies from the USA was 2.49 (95% CI: 1.88–3.29, *P* < 0.00001). Study heterogeneity was low among the studies from both these countries, which may be related to improving participant compliance and acceptance. Due to the inclusion of Shoo's study, the studies from Canada were highly heterogeneous. In summary, the data from the three countries (RR = 2.22, 95% CI: 1.69–2.91, *P* < 0.00001) showed that the incidence of NEC decreased after intervention measures were implemented, and the difference in results was statistically significant.

### 3.6 Publication bias and sensitivity analysis

**Figure 5:**
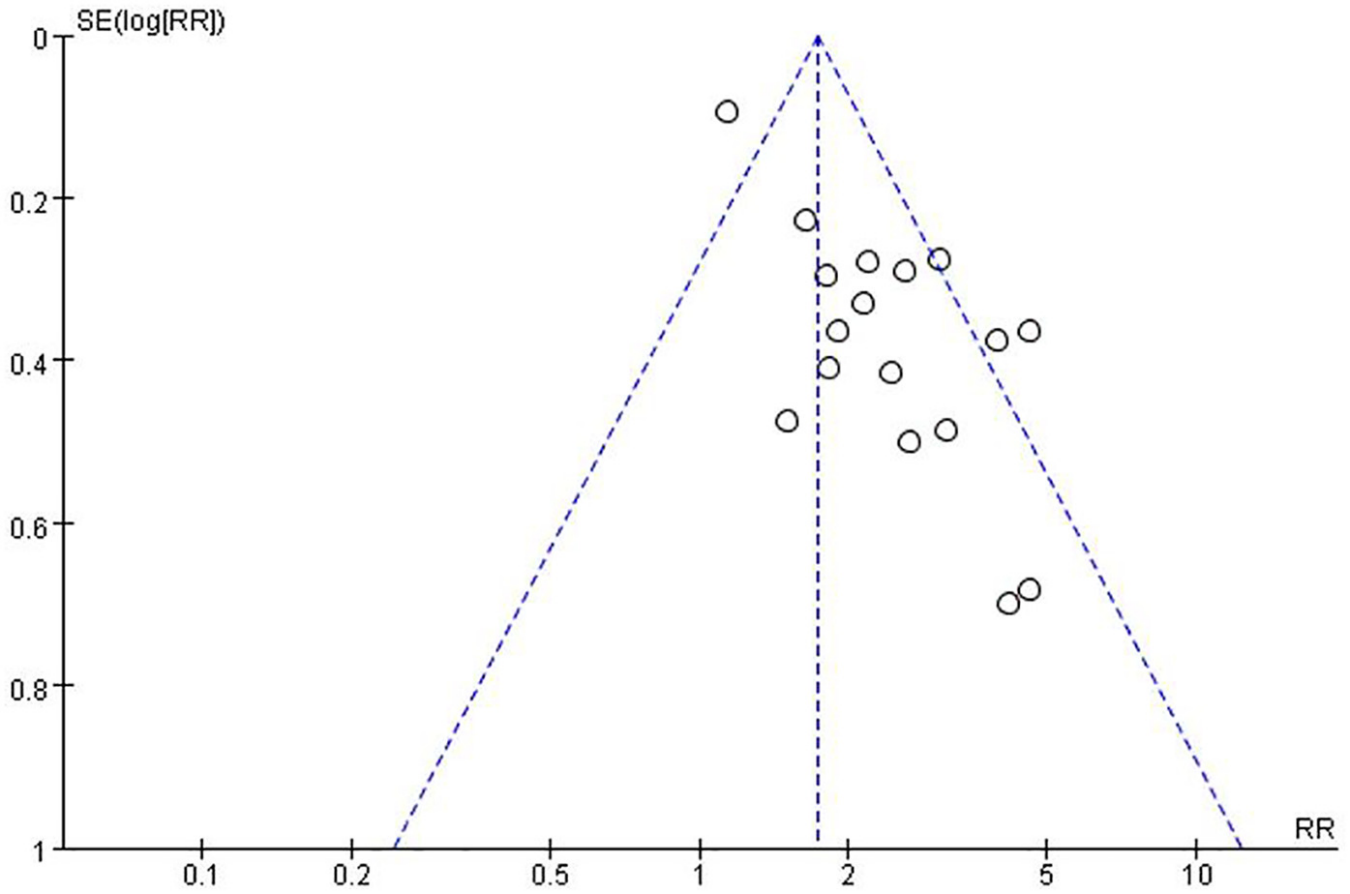
Funnel plot of publication bias for the rate of NEC.

**Figure 6:**
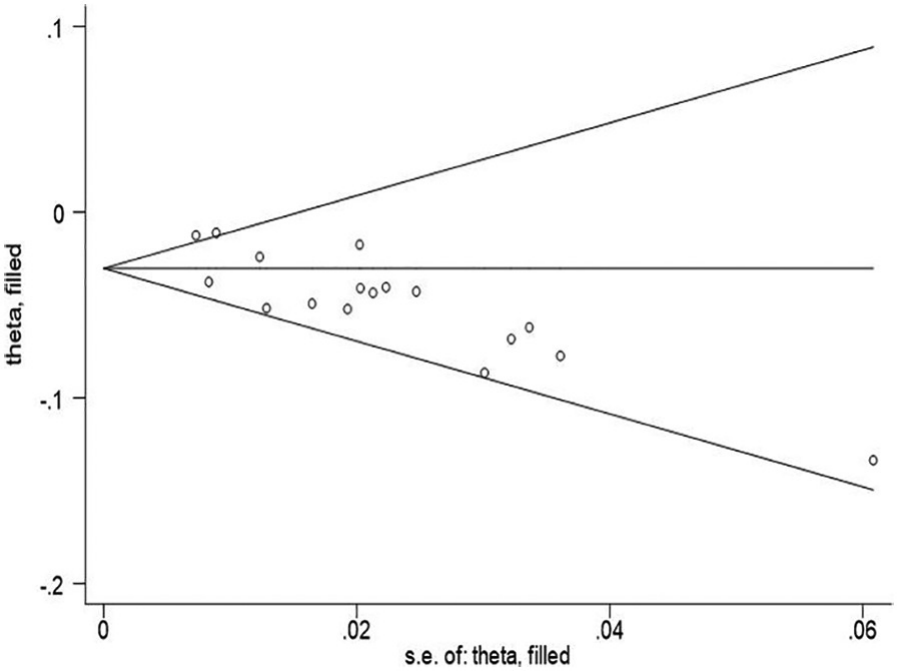
Funnel plot of publication bias for the rate of NEC after correction.

Both funnel plots and Egger’s test show publication bias. In this study, the risk of publication bias was graphed using a funnel plot, and the visual examination revealed asymmetry (Figure 5). At the same time, we used Egger’s test to show that there was also significant publication bias (*P* = 0.001 < 0.05). To assess the stability of the results, we utilized the trim and fill method to estimate the number of possible missing studies and their impact. The results of the heterogeneity test were as follows: Q = 28.948, *p* = 0.024 < 0.05 (Figure 6). There was no significant change in the results of the meta-analysis before and after trimming, and no significant effect of publication bias, indicating that our results were relatively stable.

## 4 Discussion

We conducted a systematic review and meta-analysis of published quality improvement measures involving a total of 18,314 newborns, identifying 13 QIIs aimed at reducing the incidence and severity of NEC. Most of the interventions focused on developing local evidence-based research and designing driver diagrams to facilitate the advancement and sustainability of quality improvements. Although the meta-analysis showed high heterogeneity, it also showed that the implementation of QIIs resulted in a reduction in the incidence of NEC in preterm or low-birth-weight infants in most areas. The incidence of SNEC and mortality reflect the severity of the disease (30). The results of this study showed that QIIs were positively associated with a decrease in the incidence of SNEC, and the decrease in the incidence of SNEC was associated with multiple regional interventions.

The systematic review identified the role of QIIs in reducing the incidence of NEC in NICUs. Most of the QIIs included a wide range of clinical interventions, and it is unclear which specific interventions and quality improvement methods were responsible for the change in the incidence of NEC. The same interventions may not have the same results when implemented in different regions, as they may be influenced by healthcare professionals, the culture of the organization involved in the QI, available resources, and so on (31). Therefore, when selecting which clinical interventions to include in QIIs, it is important to evaluate the potential evidence for each clinical intervention and carefully weigh the potential benefits and risks. Randomized trials of most clinical interventions may not be available. Knowing that others have successfully tested and implemented specific clinical interventions can support local change testing using the QI approach.

Evidence-based quality improvement is a system for improving processes and outcomes for patients in a clinical setting (32, 33). It is data- and evidence-driven and focuses on improving workflows to deliver high-quality care based on the strongest evidence combined with the clinician's expertise to meet the needs of a particular setting or population. Data-driven, evidence-based QI initiatives and a QI's track record can contribute to the success of QIIs (34, 35). In this study, the included study QIIs were evaluated using QI-MQCS, and the number of clinical interventions for each QIIS ranged from 1 to 8. The QI-MQCS scores showed that all the interventions were considered to be of medium to high quality, with the lowest score being 8 and 13 had scores greater than 10. Some of the NICUs continued with the QIIs after the end of the active intervention, indicating the sustainability of the QIIs.

NEC is a multifactorial disorder, the exact pathogenesis of which is incompletely understood and in which various risk factors play an important role (36, 37). A variety of factors are involved in the pathogenesis of NEC, including preterm birth, low birth weight, lack of breast milk exposure, changes in the microbiome, maternal and environmental factors, and intestinal ischemia and hypoxia. In NICU management, intake of non-steroidal drugs such as ibuprofen and indomethacin, infection, early antibiotic exposure, and PPI and H2 receptor antagonist use may directly or indirectly increase the chance of NEC. A retrospective cohort study in Kenya showed that antenatal steroid exposure, duration of mechanical ventilation, and duration of umbilical vein cannulation were three independent modifiable risk factors associated with NEC stages IIa–IIIb (38).

Gephart et al. (39) used the Grading of Assessment, Development, and Evaluation (GRADE) criteria to summarize modifiable NEC risk factors during the antenatal and intrapartum stages and early and late clinical course, grading the evidence to identify quality improvement strategies that can reduce the risk of NEC. There were differences in the content of the QIIs in this study, and some included the same clinical interventions. Some of these measures emphasize the important role of breast milk in preventing the development of NEC, such as increased breastfeeding rates and breast milk oral immunity, and it is suggested that the delivery of immune and growth factors in breast milk to the immature intestinal mucosa may promote physiological, neuroendocrine, and metabolic adaptation in very premature or very-low-birth-weight infants (40, 41). This is also supported by evidence from many randomized controlled trials (42–45).

Other measures, including clinical interventions aimed at avoiding impaired intestinal blood flow or relative intestinal hypoxia, are supported by evidence from randomized trials (46–49). These include DCC, conservative feeding during PDA treatment, and standardized blood transfusion. In addition, intestinal flora disturbances may be associated with the development of NEC (50–52), and interventions to prevent these disturbances include probiotic supplementation, antibiotic use, restriction of gastric tube use, and restriction of PPIs and H2 receptor antagonists. Some of the QIIs in this study also emphasized the importance of parental involvement, learning about the prevention of NEC, proactively offering breast milk, and participating in kangaroo care to increase the confidence of parents of preterm or very-low-birth-weight infants to maximize the benefit to the neonate. The QIIs also include standardizing the use of breast milk fortifier, standardizing the management of feeding intolerance, and limiting enteral nutrition osmolality, which may indirectly affect the occurrence of NEC.

Our meta-analysis included 18,314 infants, the largest sample to date to analyze the relationship between QIIs and NEC. The results of the meta-analysis indicated a significant association between interventions and the occurrence of NEC. Compared to previous reviews and meta-analyses, this study included more original studies, analyzed the effect of quality improvements on surgical NEC, and included additional subgroup analyses of studies from different countries. While these analyses showed that quality improvements in clinical implementation not only led to fewer newborns developing NEC but also to a reduction in the incidence of surgical NEC across countries, they need to be repeated and confirmed in more prospective cohort studies.

We also used the QI-MQCS to assess the evidence. However, our review had limitations. First, although a large number of studies were included in the review, it is possible that some studies were missed despite a rigorous search strategy and thorough manual search. Second, the interventions implemented in different NICUs were different, the implementation varied, and determining the effectiveness of a particular intervention was difficult. Third, the studies included in the meta-analysis were highly heterogeneous due to differences in intervention characteristics, implementation units, personnel, sample size, time, and outcomes.

Our review highlights that NEC can be prevented through quality improvement. However, it is essential to establish a unified and standardized approach for reporting results. We suggest that future research should focus on identifying the processes that can be effectively implemented to reduce NEC occurrence and which bundle elements represent important components. It is essential to clarify the research population, standardize the intervention measures, unify the timing of interventions, assess the sustainability of these measures, and develop a standardized protocol. Such efforts may facilitate the promotion and implementation of effective strategies across diverse regions and countries worldwide.

A significant limitation of this review is the possibility of publication bias. Since interventions do not require registration, QIIs are at a heightened risk of bias compared to randomized trials. Studies that fail to demonstrate quality improvement are generally more challenging to publish compared to effective interventions. This is a common risk of publication bias in systematic reviews. Currently, the existing tools for assessing risk of bias do not adequately address the unique characteristics associated with reporting QIIs. New tools to assess bias are needed to help readers evaluate the risk of adjusting and testing for NEC in QII reports.

## 5 Conclusions

A systematic review of the experiences of teams involved in QIs could provide valuable input to those exploring quality improvement efforts in their own field. There is now substantial evidence that the implementation of QIIs in neonatal intensive care units reduces the incidence and severity of NEC in preterm infants. Given the heterogeneity of the clinical interventions used and the variation in reporting quality, to realize the potential of incorporating QIIs into evidence-based practice, future research should focus on identifying which processes could help teams effectively reduce the incidence of NEC and which bundling elements are important.

## Data Availability

The original contributions presented in the study are included in the article/Supplementary Material, further inquiries can be directed to the corresponding authors.
